# Shining a Light on the Mechanisms of Drug Resistance to Anti‐PD‐1 Therapy in Non‐small Cell Lung Cancer

**DOI:** 10.1155/humu/7216680

**Published:** 2026-10-28

**Authors:** Hongsheng Guo, Mengmeng Jiang, Hailian Zhang, Jian Zhao, Ying Luo

**Affiliations:** ^1^ Department of Medical Oncology Tianjin Third Central Hospital/Central Hospital Tianjin University Tianjin China tju.edu.cn; ^2^ Tianjin Key Laboratory of Extracorporeal Life Support for Critical Diseases Tianjin Institute of Hepatobiliary Disease Tianjin Third Central Hospital/Central Hospital Tianjin University Tianjin China tju.edu.cn

**Keywords:** drug resistance, genome-wide association studies, immune checkpoint inhibitors, nonsmall cell lung cancer, single-cell sequencing analysis, tumor microenvironment, tumor mutation burden

## Abstract

Immune checkpoint inhibitors (ICIs) are first‐line therapies for advanced Nonsmall cell lung cancer (NSCLC), yet managing ICI resistance remains a critical, unavoidable challenge. This study identified genetic markers of anti‐PD‐1 resistance and developed an exploratory predictive model to differentiate responsive and resistant NSCLC patients. The GSE126044 transcriptomic subseries was used as the discovery cohort and included 16 pretreatment NSCLC biopsies (5 responders and 11 nonresponders). The prespecified eight‐gene score was re‐evaluated using repeated stratified out‐of‐fold prediction and was further assessed in two independent checkpoint‐blockade cohorts. The averaged out‐of‐fold AUC in GSE126044 was 0.982 (95% CI 0.931–1.000), although the eight genes had been selected before cross‐validation. External discrimination was limited in GSE135222 (AUC = 0.636, 95% CI 0.363–0.909) and GSE253564 (AUC = 0.567, 95% CI 0.230–0.903). These findings do not support stable generalization of the score across independent treatment settings. The eight‐gene score should be regarded as an exploratory checkpoint‐blockade response‐associated signature. Its potential clinical value requires validation in larger, prospectively collected cohorts with harmonized treatment and response endpoints.

## 1. Introduction

Lung cancer as one of the most frequent contributors to cancer mortality all over the world is expected to affect 28.4 million people by 2040, an increase of about 50% compared with 2020 [1]. In 2023, statistics showed that lung cancer accounted for 21% of all cancer‐related deaths [2]. Non‐small cell lung cancer (NSCLC) as one of the main types of lung cancer composes about 80%–85% of all lung cancer cases [3]. NSCLC can be further subdivided into adenocarcinoma, squamous cell carcinoma, large cell carcinoma, as well as rare types such as large cell neuroendocrine carcinoma [4, 5]. NSCLC patients are treated according to their stage, and surgery is the gold standard for patients at Stage I and II and some with Stage III tumors; Stage IV patients receive chemotherapy, palliative radiotherapy, or supportive care [6]. However, the efficacy of these traditional treatment strategies has reached a plateau, and the prognosis of patients is far from satisfactory [7].

The emergence of immune checkpoint inhibitors (ICIs) heralds a transformative era in the field of cancer treatment [8, 9]. NSCLC is one of the most sensitive cancers to ICIs, which have become the first‐line treatment for advanced NSCLC [10]. There are already several ICIs approved by the FDA for NSCLC treatment, such as Cemiplimab, Nivolumab, and Pembrolizumab targeting PD‐1, and Tremelimumab and Ipilimumab targeting CTLA‐4, as well as anti‐PD‐L1 monoclonal antibodies Atezolizumab and Durvalumab [11]. Among them, specific binding of PD‐1 inhibitors to PD‐1 will weaken the immunosuppressive regulation of T lymphocytes and allow them to be involved in the killing of tumor cells [12]. PD‐1 is expressed on macrophages, dendritic cells, B lymphocytes and NK cells in addition to T cells, indicating the efficacy of PD‐1 in reshaping the tumor immune microenvironment or even systemic antitumor immunity [13, 14]. Although ICIs including PD‐1 inhibitors have shown clinical efficacy in a subset of NSCLC patients [15], managing acquired resistance to ICI therapy is still challenging [16]. Moreover, ICI treatment has triggered a series of immune‐related adverse events, including T/B cell dysregulation, cytokine imbalance, autoantibody production, and genetic susceptibility [17, 18]. Therefore, comprehensive studies probing into the mechanisms underlying acquired resistance to ICIs could help overcome these problems. Previous studies on exploratory predictive models for anti‐PD‐1 resistance in NSCLC have demonstrated that the response rate to PD‐L1‐guided immunotherapy does not exceed 30% [19]. Moreover, single‐biomarker strategies are insufficient, highlighting the demand for additional immunotherapeutic exploratory predictive models and biomarkers for NSCLC.

Although large‐scale bulk transcriptomic and bioinformatic studies have deepened our knowledge of immunotherapy resistance in NSCLC, such bulk‐level analyses cannot resolve the complex intratumoral cellular heterogeneity that shapes treatment outcomes [20, 21]. Single‐cell sequencing represents a powerful innovative tool for deciphering intricate cell‐to‐cell interactions between tumors and the immune microenvironment, permitting systematic, high‐resolution dissection of heterogeneity linked to anti‐PD‐1 resistance [22, 23]. This study constructed a comprehensive research idea to address the problem of resistance to ICI therapy in NSCLC patients, which integrated multilevel bioinformatics methods such as immunotherapy cohorts, genome‐wide association studies (GWAS), and single‐cell data, as well as cell experiments, to comprehensively understand the response and resistance mechanism of anti‐PD‐1 therapy in NSCLC.

## 2. Materials and Methods

### 2.1. Download and Summary of Sequencing Data

The data of NSCLC samples were acquired from the NCBI (https://www.ncbi.nlm.nih.gov/) search bar with GSE126044, GSE41271, and GSE68465 as the search numbers. The data of TCGA‐LUAD cohort and TCGA‐LUSC cohort in TCGA were downloaded from the UCSC Xena (http://xena.ucsc.edu/) data management platform. We used principal component analysis (PCA) to determine that the expression profiles between the two cohorts representing different NSCLC types were inherently different, and there was no obvious batch effect, so the expression profiles of the two cohorts were directly integrated, and each sample had complete survival information and complete copy number variation (CNV), single nucleotide polymorphisms (SNP), methylation, and transcriptome data.

### 2.2. Screening of Genes Associated With Anti‐PD‐1 Therapy Resistance

RNA‐expression data were obtained from GSE126044, a transcriptomic subseries of GSE126045. Sixteen pretreatment NSCLC biopsies were available, including five responders and 11 nonresponders. Eleven samples were fresh biopsies and five were FFPE samples. Differential expression in the original discovery workflow was defined by Benjamini–Hochberg‐adjusted *p* < 0.05 and NR‐versus‐R log2 fold change ≥ 3. To assess the effect of this stringent large‐effect prioritization threshold, we repeated the DEG‐survival‐gene intersection using log2 fold‐change cutoffs of 1, 1.5, 2, 2.5, and 3. We additionally performed post‐hoc ashr shrinkage using the available DESeq2 maximum‐likelihood estimates and standard errors. Because the original DESeq2 DDS object was not retained, this analysis was not a rerun of DESeq2 lfcShrink.

### 2.3. ceRNA Network Analysis of Candidate Genes

Cancer‐related miRNAs were obtained from the Human microRNA Disease Database (HMDD) [24]. The miRNAs targeting the candidate genes related to anti‐PD‐1 treatment resistance were extracted from miRWalk database [25], and then the intersection of miRNAs with tumor‐related miRNAs was taken. The lncRNAs acting on the intersection miRNAs were predicted using the ENCORI database, resulting in ceRNA networks of lncRNA‐miRNA‐candidate mRNAs.

### 2.4. Analysis of Transcriptional Regulation of Candidate Genes

We attempted to reconstruct the motif annotation file according to motifs‐v10nr to integrate the regulatory information contained in hg38_10kbp_up_10kbp_down_full_tx_v10_clust.genes_vs_motifs.rankings.feather. The RcisTarget package was used to predict the upstream transcription factors (TFs) based on the candidate genes, and motif enrichment analysis was performed to construct the TF regulatory network.

### 2.5. GWAS of Candidate Genes

We accessed the Gene Atlas database to find the GWAS data of candidate genes related to PD‐1 resistance, identified the significant SNPs related to PD‐1 resistance, and then analyzed the genetic regions of these significant SNPs, including whether they were within known resistance‐associated genes or their regulatory regions.

### 2.6. Construction and Validation of Anti‐PD‐1 Resistance Score Model

The eight‐gene feature set was fixed before model re‐evaluation. Internal performance was assessed using 20 repeats of stratified five‐fold outer cross‐validation. Within each outer training partition, gene‐wise centering and scaling were estimated from training samples only, and alpha and lambda were selected by four‐fold internal cross‐validation. Held‐out samples were used exclusively for out‐of‐fold prediction. ROC AUC and DeLong 95% confidence intervals, PR‐AUC, sensitivity, specificity, accuracy, and balanced accuracy were reported. Because the eight genes had originally been selected using the full discovery cohort, this procedure evaluates the fixed feature set and does not eliminate feature‐selection leakage.

The fixed model coefficients were applied without refitting to GSE135222 and GSE253564. In GSE135222, durable clinical benefit was reconstructed as progression‐free survival > 180 days. In GSE253564, the analysis was restricted to pretreatment RNA‐seq samples from the Durvalumab‐plus‐SBRT arm, and major pathologic response was defined as at least 90% tumor regression. The original model object did not retain discovery‐cohort centering and scaling parameters; therefore, gene‐wise standardization was performed within each external cohort following the delivered workflow. This preprocessing limitation was considered when interpreting external performance. Cohort‐derived Youden thresholds were treated as exploratory.

### 2.7. Genetic Variation Analysis

The list of tumor mutation genes was downloaded from the Cancer Gene Census (CGC) of COSMIC database (https://cancer.sanger.ac.uk/cosmic), and the “maftools” package was used to plot the waterfall map of tumor driver gene mutations in the TCGA data set of anti‐PD‐1 treatment response group and nonresponse group. Differential mutation events were determined by Fisher′s exact test for the mutation information of the response and sensitive groups. Homology recombination defect (HRD) score and neoantigen load information were obtained from previous publications, and rank sum test was adopted to determine the differences of these indicators between the resistant group and the sensitive group. The methylation data of NSCLC obtained from TCGA were imported into ChAMP package for reading. Methylation analysis was performed to confirm the methylation sites that showed significant differences between the anti‐PD‐1 treatment‐sensitive group and the anti‐PD‐1 resistance group, and the intersection with DEGs was performed to determine the DEGs that may be driven by methylation. Their annotated functional pathways were then determined by performing enrichment analysis.

### 2.8. Analysis of Pathway Characteristics

The hallmark gene set and the “GOBP T CELL MEDIATED IMMUNE RESPONSE TO TUMOR CELL” gene set were obtained from MSigDB. The enrichment scores of hallmark gene sets were compared between the anti‐PD‐1 sensitive group and the anti‐PD‐1 resistant group to confirm the differences in the enrichment scores of related pathways. Enrichment scores were calculated using the “GSVA” package, where the method was set to ssGSEA and the data distribution was Poisson.

### 2.9. Immune Cell Infiltration and Immune Molecule Expression Analysis

CIBERSORTx, EPIC, TIMER, quanTIseq, MCPcounter, xCell and TIMER 2 based on transcriptome algorithms were used to evaluate the infiltrating abundance of immune cells in the tumor immune microenvironment. T‐cell receptor (TCR) richness was calculated following a past study [26]. Immune checkpoint genes with clear inhibitory and activating effects were acquired from ImmPort database, and their expression heat map was drawn using the “heat map” package, and the correlation network between differential immune checkpoints and DEGs between groups was constructed by Cytoscape.

### 2.10. Processing of Single‐Cell Sequencing Data of NSCLC

Single‐cell data of five NSCLC tumor samples were collected from the literature published by Lambrechts et al. [27]. The R package “Seurat (v4.7.1.3)” was used for quality control and subsequent clustering, dimensionality reduction analysis of the data. All functions ran with default parameters. Known cell type markers derived from the literature were employed to classify cell types.

### 2.11. Analysis of Communication Between Cells

The communication network between cells was generated by the R package “CellChat (v1.5.0)” [28]. After creating the CellChat object, the reference database was the secreted signaling pathways and the default parameters were used to screen putative interaction pairs.

### 2.12. Two‐Sample Mendelian Randomization Analysis of Gut Microbiota and Circulating Protein Levels

We performed an exploratory two‐sample MR reconstruction using publicly available summary statistics. The exposure was genetically predicted *Bilophila wadsworthia* abundance from the Dutch Microbiome Project (GCST90027814; *n* = 7738 participants of European ancestry), and the direct outcome was circulating ITGB6 protein abundance from the AGES‐Reykjavik serum pQTL study (GCST90089794; *n* = 5366 participants of Icelandic/European ancestry). The GEO and TCGA transcriptomic and clinical datasets were not used as outcome GWAS datasets. The NMP study used shotgun metagenomic profiling, imputed genome‐wide genotypes, and a linear mixed model. The AGES study used SomaScan/SOMAmer serum protein measurements, Illumina hu370CNV and Global Screening Array genotypes, HRC r1.1/Minimac3 imputation, postimputation R2 ≥ 0.7 and MAF ≥ 0.01, and linear regression adjusted for age, sex, five genetic principal components, and genotyping platform. The complete default NMP covariate set was not independently recoverable from the accessible public summary metadata and was not inferred.

A relaxed locus‐wide threshold of *p* < 5 × 10^−6^ was considered because few microbiota instruments were available at *p* < 5 × 10^−8^. The two SNPs explicitly retained in the supplied original MR result (rs11768363 and rs10276776) were verified in both public summary files and used for reconstruction; the original LD‐clumping output was unavailable, and we therefore do not claim a de novo reproduction of the clumping step. Alleles were harmonized to the same effect allele. Both instruments were nonpalindromic, neither required a proxy, and no ambiguous SNP was retained. Instrument strength was calculated as F = (beta_exposure/SE_exposure)^2^. The primary estimate used inverse‐variance weighting. Cochran′s Q, manual leave‐one‐out, and Steiger directionality analyses were conducted. MR‐Egger, weighted‐median/mode estimates, the Egger intercept, and MR‐PRESSO were not considered reliable with two forward instruments. Reverse MR used one independent trans‐pQTL for ITGB6 and the Wald‐ratio method. No multiplicity‐adjusted *p* value was inferred for this isolated reconstruction because the original 44 × 8*p*‐value matrix was unavailable.

### 2.13. Statistical Analysis

All bioinformatics data were performed by R software for statistics and analysis, and differences between the two groups of variables were calculated by the Wilcoxon rank‐sum test. For *p* values, values < 0.05 were considered to be statistically significant.

## 3. Results

### 3.1. Potential Genes and Molecular Mechanisms Driving Resistance to Anti‐PD‐1 Therapy in NSCLC

Through differential expression analysis between anti‐PD‐1 responders and nonresponders using the GSE126044 transcriptomic subseries (SuperSeries GSE126045), 42 genes were significantly upregulated in the resistant group relative to the sensitive group (Figure 1A). A total of 2388 survival‐related genes were screened from TCGA across both major NSCLC histological subtypes. We further explored overlapping genes between resistance‐associated DEGs and survival‐related genes across successive log₂ fold‐change cut‐offs (1, 1.5, 2, 2.5, and 3), which yielded 17, 15, 13, 10, and 8 overlapping genes, respectively (Table S1). Nine additional candidate genes were identified at log₂FC ≥ 1: *COL4A3*, *SLC26A5*, *FAM111B*, *KRT80*, *PAWR*, *FRMD5*, *EMP2*, *TMC7*, and *ZNF544*. Among these tested thresholds, we finally adopted the strictest cutoff of log₂FC ≥ 3, obtaining eight genes that were both anti‐PD‐1‐resistance‐associated and NSCLC survival‐related genes. These eight genes were regarded as potential drivers of anti‐PD‐1 resistance in NSCLC (Figure 1B,C). All eight prespecified genes retained positive post‐hoc ashr posterior means (range 2.759–5.515), with posterior intervals excluding zero. Because the thresholds were nested and all analyses reused the discovery‐cohort data, these findings do not constitute independent validation. Next, we explored the upstream molecules of eight potential genes that may regulate resistance to anti‐PD‐1 therapy in NSCLC, including TFs and ceRNAs. A total of 1328 miRNAs targeting anti‐PD‐1 therapy resistance candidate genes extracted from miRWalk database were intersected with 497 tumor‐related miRNAs in HMDD to obtain six miRNAs that may target anti‐PD‐1 therapy resistance potential genes (Figure 2A). The lncRNAs that act on these six miRNAs were predicted by ENCORI database, thereby forming a ceRNA network of lncRNA‐miRNA‐anti‐PD‐1 treatment resistance potential genes (Figure 2B). Motif enrichment analysis showed that the potential genes driving anti‐PD‐1 therapy resistance in NSCLC may be regulated by TFs, including CUX1, ZNF485, and ZNF577 (Figure 2C).

**Figure 1:**
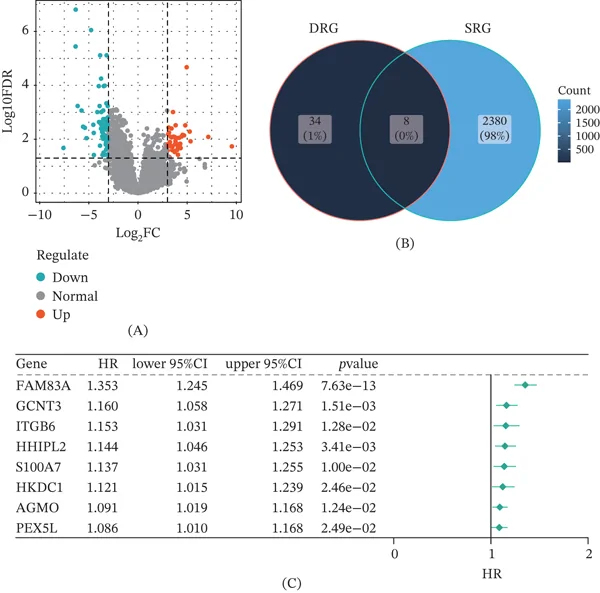
Potential genes and molecular mechanisms driving resistance to anti‐PD‐1 therapy in NSCLC. (A) DEGs in the resistant group compared with the sensitive group in the GSE126045 dataset. (B) Venn diagram of anti‐PD‐1 therapy resistance associated genes and NSCLC survival associated genes. (C) Prognostic forest plot of eight potential genes driving resistance to anti‐PD‐1 therapy in NSCLC.

**Figure 2:**
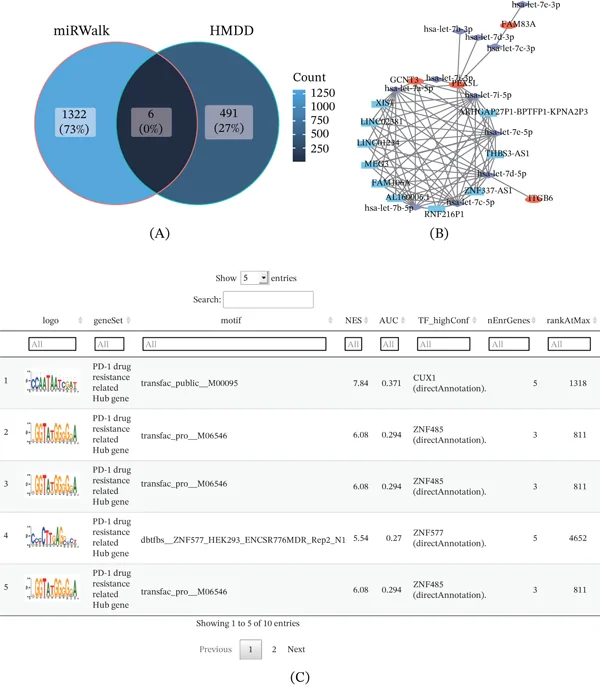
Molecular mechanisms of potential genes driving resistance to anti‐PD‐1 therapy in NSCLC. (A) Venn diagram of miRNA extracted from miRWalk database targeting candidate genes for anti‐PD‐1 therapy resistance and tumor‐associated miRNA in HMDD. (B) ceRNA network of lncRNA‐miRNA‐anti‐PD‐1 treatment resistance potential genes. (C) Upstream TFs of potential genes driving resistance to anti‐PD‐1 therapy in NSCLC.

### 3.2. Genome‐Level Regulatory Analysis of Potential Genes Involved in Anti‐PD‐1 Therapy Resistance

Autosomal SNPs were examined by multivariable association tests. The plotted Quantile‐quantile (QQ) plots showed that multiple SNPs were associated with the levels of potential genes for anti‐PD‐1 treatment resistance at the GWS level (Figure 3A). Manhattan plot highlights three susceptibility loci that may have genome‐wide significance for NSCLC (Figure 3B). For the potential genes of anti‐PD‐1 treatment resistance, we also identified the corresponding pathogenic regions of SNPs (Figure 3C).

**Figure 3:**
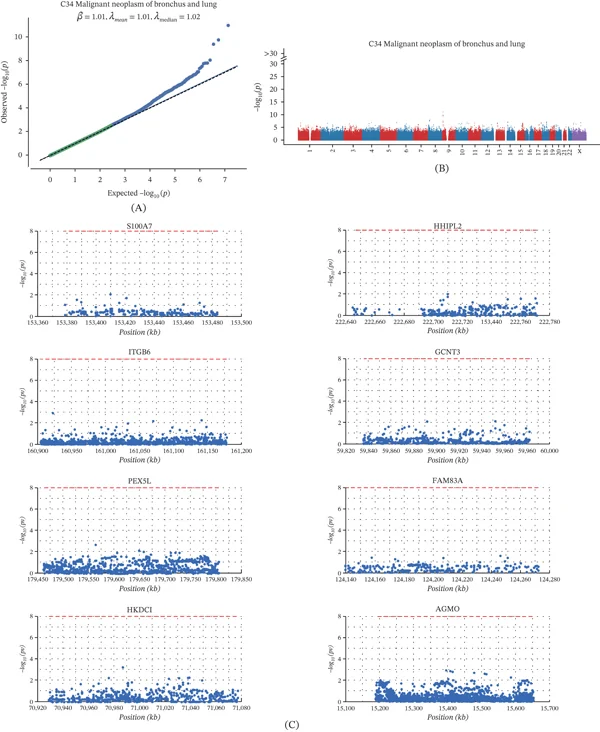
Genome‐level regulatory analysis of potential genes involved in anti‐PD‐1 therapy resistance. (A) QQ plots of GWAS data from the Gene Atlas database, the *x*‐axis indicates the negative log‐scale of ideal *p*‐values of each SNP, whereas the *y*‐axis represents the negative log‐scale of the observed *p*‐values. (B) Manhattan plots of pathogenic regions across the genome. (C) SNP pathogenic regions of potential genes for anti‐PD‐1 treatment resistance.

### 3.3. Construction and Evaluation of Anti‐PD‐1 Therapy Resistance Score Model

GSE126044 comprised 16 pretreatment NSCLC biopsies, including 5 responders and 11 nonresponders. Repeated stratified evaluation of the fixed eight‐gene feature set yielded an averaged out‐of‐fold AUC of 0.982 (95% CI 0.931–1.000), sensitivity of 0.909, specificity of 1.000, and accuracy of 0.938 at the cohort‐derived Youden threshold (Figure 4A,B). Because gene selection preceded cross‐validation, these results represent conditional internal performance rather than unbiased end‐to‐end validation. In GSE135222 (*n* = 27; DCB *n* = 7 and NDB *n* = 20), the fixed score yielded an AUC of 0.636 (95% CI 0.363–0.909) (Figure 4C). The score did not differ significantly between DCB and NDB groups (Wilcoxon *p* = 0.306) (Figure 4D), and the continuous‐score Cox model was not significant (HR = 4.387, 95% CI 0.4 65–41.391; *p* = 0.1966) (Figure 4E). In GSE253564 (*n* = 16; MPR *n* = 10 and no_MPR *n* = 6), the AUC was 0.567 (95% CI 0.230–0.903), the PR‐AUC was 0.567, and the score difference was not significant (Wilcoxon *p* = 0.7042) (Figure S1A,B). Thus, the two small external cohorts did not provide strong evidence of generalizable discrimination.

**Figure 4:**
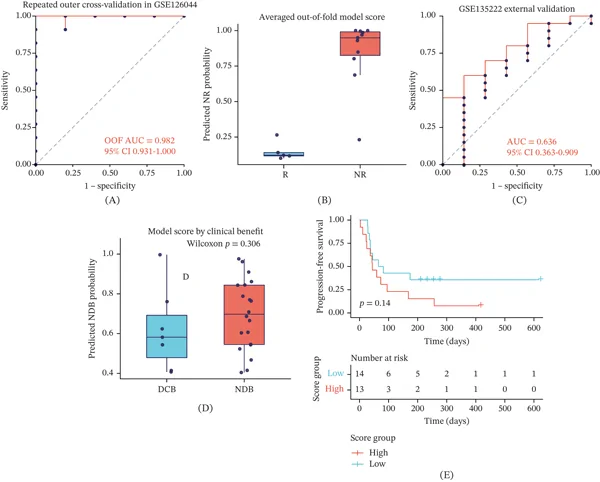
Internal and external evaluation of the eight‐gene PD‐1/PD‐L1 blockade response‐associated score. (A) Repeated stratified out‐of‐fold ROC curve in GSE126044. (B) Averaged out‐of‐fold scores in responders and nonresponders. (C) ROC curve in GSE135222, with durable clinical benefit defined as PFS > 180 days. (D) Score distributions in the DCB and NDB groups. (E) Kaplan–Meier curves for PFS according to the cohort‐median score. Cohort‐derived thresholds and groups are exploratory. The GSE126044 estimate is conditional on eight genes selected before cross‐validation.

### 3.4. Characteristics of Genetic Variants in NSCLC Patients Based on Anti‐PD‐1 Therapy Resistance Score Model

We mapped the mutation profiles of PD‐1 sensitivity and resistance groups in NSCLC based on anti‐PD‐1 therapy resistance score model, including but not limited to TP53, CSMD3, MUC16, LRP1B and other high‐frequency mutated genes (Figure 5A). Moreover, the tumor mutation burden (TMB), and HRD score in the sensitive group were noticeably higher than the resistant group (Figure 5B,C). p.G12V, p.G12C, p.G12D, p.G12A, and p.G13C mutations in KRAS gene were not significantly correlated with the anti‐PD‐1 resistance score (Figure 5D). From the perspective of CNV, the probability of copy number amplification and copy number deletion in the resistant group was significantly lower than that in the sensitive group (Figure 5E). The methylation analysis and differential expression analysis of TCGA‐NSCLC sensitive group and drug resistance group found 2178 upregulated methylation sites, 801 downregulated methylation sites, 1663 downregulated genes and 743 upregulated genes (Figure 5F). A total of 180 DEGs whose expression may be driven by methylation were identified by Venn diagram, including 140 genes that may be low expression because of hypermethylation and 40 genes that may be high expression because of hypomethylation (Figure 5G). Among them, the upregulated genes in the drug resistance group were mainly significantly enriched in coagulation and metabolism‐related processes, and the down‐regulated genes were related to growth (Figure 5H,I).

Figure 5Characteristics of genetic variants in NSCLC patients based on anti‐PD‐1 therapy resistance score model. (A) Mutational waterfall plots of PD‐1 sensitivity and resistance groups for NSCLC based on the anti‐PD‐1 therapy resistance score model. (B) TMB differences between resistant and sensitive groups in TCGA‐NSCLC. (C) Differences in HRD scores between resistant and sensitive groups in TCGA‐NSCLC. (D) Changes in anti‐PD‐1 treatment resistance prediction scores for different mutation sites within the KRAS gene in TCGA‐NSCLC. (E) CNV differences between resistant and sensitive groups in TCGA‐NSCLC. (F) Volcano map of differential methylation between resistant and sensitive groups in TCGA‐NSCLC. (G) Venn diagram for joint analysis of methylation sites and gene expression. (H,I) Pathway enrichment bubble plots of up‐regulated and down‐regulated genes in the resistance group, respectively.

**Figure:**
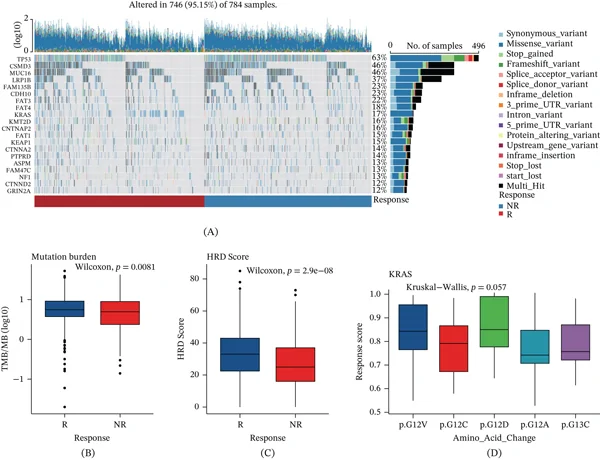

**Figure:**
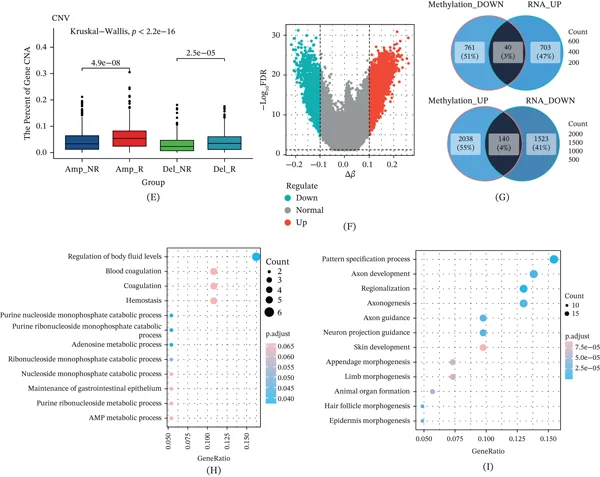

### 3.5. The Potential Mechanism of PD‐1 Resistance Was Revealed Based on Anti‐PD‐1 Drug Resistance Score Model

We explored the aberrant activity of 50 pathways in the hallmark gene set between the sensitive and resistant groups as defined by the anti‐PD‐1 treatment resistance score model. Compared with the sensitive group, apoptosis, KRAS signaling, TGF*β* signaling, metabolism and important components of the body′s immune defense mechanisms, including complement, inflammatory response, interferon alpha/gamma response, were highly active in the resistant group, while oncogenic pathways including DNA repair that induces cancer progression, PI3K‐AKT mTOR signaling, unfolded protein response, G2M checkpoint, mitotic spindle, Wnt‐*β*‐catenin signaling were absent in the drug resistant group (Figure 6A). To analyze the mechanism underlying anti‐PD‐1 resistance, we obtained T cell‐mediated tumor immune response (TMTI) gene set from MSigDB, and then analyzed the interaction between metabolic and immune‐related pathways and TMTI in the resistant group and the sensitive group, respectively. TMTI was positively correlated with multiple immune‐related pathways in the sensitive group and positively correlated with multiple metabolic regulatory pathways in the resistant group (Figure 6B,C). Therefore, immune dysfunction and abnormal metabolism may be important reasons for resistance to anti‐PD‐1 treatment.

**Figure 6:**
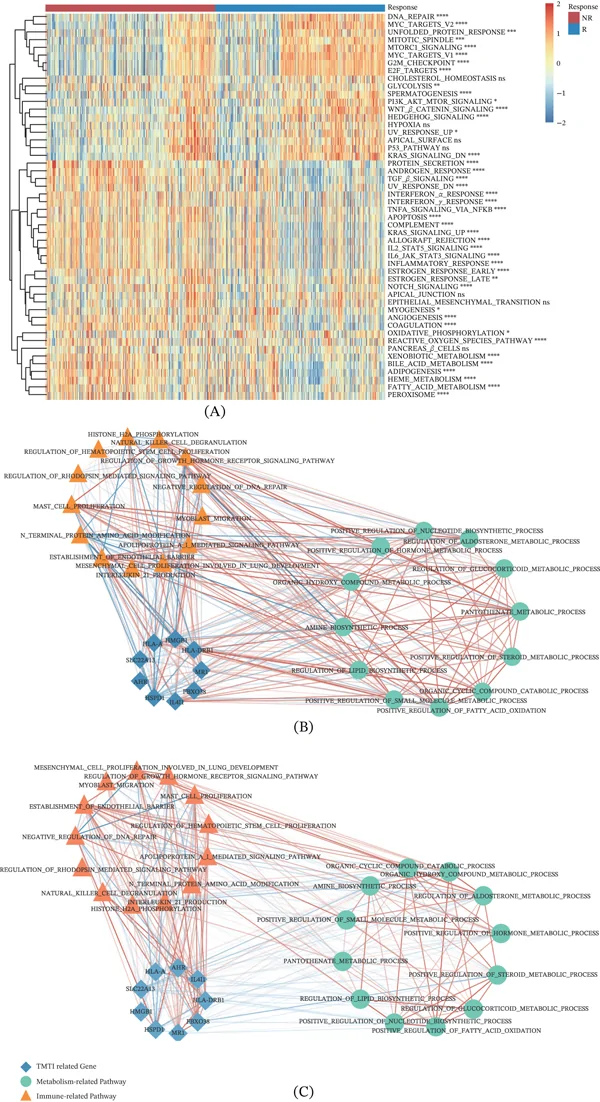
The potential mechanism of PD‐1 resistance was revealed based on anti‐PD‐1 drug resistance score model. (A) The heat map shows the enrichment scores of 50 pathways in the hallmark gene set in the sensitive group and the resistant group defined by the anti‐PD‐1 treatment resistance score model. (B,C) Interaction analysis of genes in TMTI with metabolic and immune‐related pathways in the drug resistant group and the sensitive group.

### 3.6. Immune Microenvironment Pattern Mediated by Anti‐PD‐1 Therapy Resistance Score Model

The differences in the activity of immune pathways between the anti‐PD‐1 resistant group and the anti‐PD‐1 sensitive group inspired us to further investigate the immune microenvironment patterns in the two groups. According to the seven algorithms for evaluating the abundance of immune cell infiltration, the immune surveillance and immune killing function cells, including B cells, NK cells, CD4+ T cells, etc., showed higher activity in the sensitive group. The cells that promote immune escape, including Tregs, M2 macrophages, and cancer associated fibroblast, showed high activity in the resistant group (Figure S2A,B,C,D,E,F,G).

The results of seven immune cell infiltration assessment methods showed that CD8^+^ T cells in the drug‐resistant group lacked infiltration activity (Figure S3A). Moreover, heat map analysis revealed altered immune parameters including TIP cancer antigen Presentation 1, MHC Class I, MHC Class II, and chemokine receptor between the drug‐resistance group and the sensitive group (Figure S3B). TCR richness did not show significant differences between the resistant and sensitive groups (Figure S3C). Distinct expression patterns of immune‐checkpoint genes were observed between the two groups (Figure S3D). We found interaction networks composed of immune genes and DEGs in the resistant group, and most immune genes showed negative correlation with DEGs (Figure S3E).

### 3.7. Intratumor and Intertumor Heterogeneity of Anti‐PD‐1 Therapy Resistance

We also investigated the characteristics of resistance to anti‐PD‐1 therapy at the single‐cell level. We identified 17 cell types by clustering and annotation of 33,694 cells in NSCLC, including airway epithelial cells, CD4^+^ T cells, cancer cells, fibroblasts, AT2 cells, dendritic cells, CD8^+^ T cells, erythroblasts, AT1 cells, B cells, granulocytes, Langerhans cells, endothelial cells, macrophages, mast cells, NK cells, Tregs (Figure S4A). There was great heterogeneity in cell composition between different patients (Figure S4B). In NSCLC, potential drivers of resistance to anti‐PD‐1 therapy were mainly expressed on airway epithelial cells and cancer cells (Figure S4C). In terms of distribution, immune cells such as T cells, macrophages, B cells mostly represented the major enriched populations in PD‐1 treatment‐sensitive patients. Cancer cells, AT cells, airway epithelial cells were the main enriched groups in resistant patients (Figure S4D,E).

### 3.8. Intercellular Communication Analysis Reveals the Resistance Mechanism of Anti‐PD‐1 Treatment

We explored the mechanisms of resistance to anti‐PD‐1 therapy by analyzing the molecular interactions that mediate cell‐to‐cell communication. We found that CD8+ T cells in the sensitive group had more frequent communication with other cells in the tumor microenvironment than those in the resistant group (Figure S5A). By comparing the interaction strength of each signaling pathway, specific environmental signaling pathways between the anti‐PD‐1 therapy resistant and sensitive groups were identified. In the sensitive group, the vast majority of signaling pathways were activated, including FGF, EGF, VEGF, and others. ANNEXIN signaling pathway was especially active in the resistant group (Figure S5B). In the anti‐PD‐1 treatment resistant group, SPP1 was specifically secreted by cancer cells and specifically supported communication with immune cells by interacting with (ITGAV+ITGB5), (ITGAV+ITGB1), and (ITGAV+ITGB6). In the resistant group, CXCL16‐CXCR6 was the ligand‐receptor that specifically supported the action of cancer cells on CD8^+^ T cells, whereas CD8^+^ T cells acted on cancer cells through IFNG‐ (IFNGR1 + IFNGR2) transduction. In the resistant group, macrophages have specific effects on cancer cells through SPP1‐(ITGAV + ITGB6), SPP1‐(ITGAV + ITGB1), and RETN‐CAP1 (Figure S5C,D).

### 3.9. Exploratory Two‐Sample MR Analysis of Gut Microbiota and Circulating Protein Levels

The two instruments had F statistics of 21.59 and 25.04. The two‐SNP IVW estimate for genetically predicted *B. wadsworthia* abundance and circulating ITGB6 protein level was beta = −0.329 (95% CI −0.589 to −0.069; nominal *p* = 0.01326) (Figure S6A,B). Cochran′s Q test yielded *p* = 0.4065. Manual leave‐one‐out estimates showed consistent directional effects after removing either SNP (Figure S6C). The Steiger test supported the forward orientation for these instruments (*p* = 0.01789). Reverse MR using one ITGB6 trans‐pQTL yielded beta = 0.111 (95% CI 0.014–0.208; nominal *p* = 0.02420) (Figure S6D). Because the original 352‐test matrix was unavailable, no BH‐adjusted *q* value was reported. Nominal evidence in both directions did not establish a unique causal direction.

## 4. Discussion

The originally reported training AUC of 1.00 was optimistic because feature selection, model construction, and evaluation used the same small discovery cohort. Although the fixed eight‐gene set showed high conditional out‐of‐fold discrimination, performance was lower and imprecise in two independent cohorts with different treatment settings and endpoints. The score should therefore be interpreted as an exploratory PD‐1/PD‐L1 blockade response‐associated signature rather than a robust clinical predictor.

FAM83A overexpression was related to early lymph node metastasis and poor outcome among LUAD patients [29]. It is significantly negatively related to PD‐L1 expression and is regulated by multiple TFs, which may induce drug resistance through immunosuppression [30]. The upstream ceRNA mechanisms regulating FAM83A in lung cancer include circMIIP/miR‐766‐5p [31] and Circ‐ZKSCAN1/miR‐330‐5p [32]. GCNT3 was targeted and regulated by LINC00511/miR‐195‐5p in lung adenocarcinoma [29]. HHIPL2 mRNA is expressed in pancreatic cancer and lung cancer [33]. In gastric cancer, the frequency of HHIPL2 copy number gain was 25%, and the amplified HHIPL2 gene at 1q41‐q43.1 region showed the highest copy number gain‐related overexpression in gastric cancer [34]. A recent study in HCC showed that HKDC1 promoted tumor immune evasion in a CD8+ T cell‐dependent manner through STAT1/PD‐L1 activation in tumor cells [35]. Mechanistically, HKDC1 is regulated by HOXC‐AS2/miR‐876‐5p transduction to regulate endometrial cancer progression in the hyperglycemic TME [36]. In colorectal cancer, TGB6 expression has also been found to be an immune evasion strategy, suppressing antitumor immune responses and resistance to ICI therapy through the activation of latent TGF‐*β* [37]. SNP rs9825224 in PEX5L was also shown to be significantly associated with NSCLC survival [38]. The study by Liu et al. demonstrated that upregulation of S100A7 in lung squamous cell carcinoma leads to ICIs resistance, and targeted inhibition of S100A7 may enhance the efficacy of ICI by generating an inflammatory phenotype [39]. These studies stimulated our search for genetic variants and molecular mechanisms in eight potential genes that drive resistance to anti‐PD‐1 therapy in NSCLC. We identified SNP pathogenic regions of each potential gene driving anti‐PD‐1 therapy resistance in NSCLC and provided ceRNA networks of lncRNA‐miRNA‐anti‐PD‐1 therapy resistance potential genes. In terms of transcriptional regulation mechanism, the potential genes driving anti‐PD‐1 therapy resistance in NSCLC may also be regulated by TFs, including CUX1, ZNF485, and ZNF577, which has not been reported previously.

For the anti‐PD‐1 treatment resistance scoring model we established, which could effectively classify patients into anti‐PD‐1 treatment sensitive group and resistance group. Of particular interest, the drug‐resistant group showed overexpression of potential resistance driver genes, lower TMB, and active infiltration of immunosuppressive cells (Tregs, M2 macrophages, and cancer associated fibroblasts) compared with the sensitive group. High TMB was always selected to benefit from ICB treatment including anti‐CTLA‐4, anti‐PD‐1, and anti‐PD‐L1 [40]. Cancer cells acquire immune escape through the induction and recruitment of immunosuppressive cells and upregulating the expressions of different immunosuppressive molecules [41, 42]. This also explains the poor treatment of anti‐PD‐1 in the drug‐resistant group. Additionally, consistent with published studies, the resistant group also showed lower CNVs than the sensitive group, suggesting that genomic stability of tumors may play a role in anti‐PD‐1 therapy.

From the results of single‐cell data analysis, the potential driver of resistance to anti‐PD‐1 therapy was increased in epithelial cells and cancer cells, which may reveal a unique role for these two subsets in resistance to anti‐PD‐1 therapy, suggesting that individualized treatment strategies may need to take into account cellular heterogeneity within the tumor [43]. Effective anti‐PD‐1 immunotherapy is linked with the presence of polyclonal CD8+ T cells in the tumor and blood [44]. We found that CD8+ T cells in the resistant group lacked communication with other cells in the tumor microenvironment, and cancer cells interacted with CD8+ T cells through CXCL16‐CXCR6 transduction. According to Karaki et al., in lung tumors, CXCR6 deficiency impairs CD8 resident memory T‐cell recruitment [45]. Histologically, CXCL16 and CXCR6 were coexpressed in human primary lung cancer tissues and mediated lung cancer cell viability and invasion in vitro [46]. Thus, this mechanism by which cancer cells act on CD8+ T cells may also explain why PD‐1 inhibitors do not respond well in some patients. In addition, pathway‐enrichment analysis also revealed prominent alterations in TGF‐*β* signaling and metabolic pathways [47]. On the one hand, substantial evidence has demonstrated that TGF‐*β* regulates cancer‐cell proliferation and remodels the tumor microenvironment, and it is linked to primary resistance to PD‐1 blockade within the NSCLC tumor microenvironment. On the other hand, immune cells are also involved in tumor progression via metabolic reprogramming, with coordinated dysregulation of multiple metabolic pathways (nucleotide, amino acid, lipid, and glucose metabolism) observed in NSCLC [48]. Taken together, the effects of the eight‐gene signature may be associated with TGF‐*β*‐mediated stromal transformation and metabolic rewiring, which jointly promote resistance to anti‐PD‐1 immunotherapy in NSCLC. Nevertheless, this mechanistic model remains largely our hypothesis.

As an important part of the human microbiome, intestinal flora has been proven to play an indispensable role in a variety of physiological and pathological processes. There is a complex interaction between intestinal flora and host, which not only participates in the digestion of food and the absorption of nutrients, but also plays an important role in the development and regulation of the immune system. Studies have shown that intestinal flora can affect the host immune system through a variety of ways, such as regulating the differentiation, activation and function of immune cells, and affecting the secretion of cytokines. This strong link between the gut microbiota and the immune system has led researchers to wonder whether it might be related to the efficacy of tumor immunotherapy. From a translational perspective, the eight‐gene signature is unlikely to replace established clinical predictors such as PD‐L1 expression and Eastern Cooperative Oncology Group (ECOG) performance status. Nevertheless, combining this model with conventional clinical variables may facilitate improved patient stratification for anti‐PD‐1 therapy and yield more refined risk subgroups. This remains a speculative hypothesis at this stage. Future studies should evaluate whether integrating the eight‐gene score with PD‐L1 and ECOG status could enhance predictive performance compared with clinical indicators alone.

The exploratory MR reconstruction suggested a nominal association between genetically predicted *B. wadsworthia* abundance and circulating ITGB6 protein levels. Circulating protein abundance does not directly represent ITGB6 expression in lung tumor tissue or the tumor microenvironment. Moreover, sparse instruments, an unavailable 44 × 8 multiplicity context, and a nominal reverse‐MR signal preclude a unique directional claim or a clinical checkpoint‐blockade effect. The finding should be regarded as hypothesis‐generating and requires independent genetic, clinical, and experimental validation.

This study has certain limitations. The discovery cohort contained only 16 samples and five responders, and the eight genes were selected before cross‐validation. The external cohorts were small, clinically heterogeneous, and required cohort‐wise standardization because discovery‐scaling parameters were unavailable. Although we observed lower TMB in the resistant group, we did not assess the correlation between TMB and the expression levels of the eight signature genes. Therefore, we cannot exclude the possibility that the predictive performance of our model merely reflects underlying TMB differences across patients. Future independent datasets with paired gene‐expression and TMB data will be needed to evaluate the correlation between signature‐gene expression and TMB, and to disentangle the predictive value of our eight‐gene score from TMB‐related effects. The MR reconstruction included two forward instruments and one reverse trans‐pQTL; the original 44 × 8*p*‐value matrix and a reproducible LD‐clumping record were not available. Accordingly, neither stable clinical prediction nor a definitive microbiota‐ITGB6 causal pathway can be inferred.

## 5. Conclusion

In summary, this study provided new insights into the molecular mechanisms of anti‐PD‐1 resistance and potential exploratory predictive models to guide anti‐PD‐1 therapeutic interventions, providing a basis for in‐depth investigation of immune escape mechanisms and development of novel approaches to overcome PD‐1 resistance.

**Nomenclature**
AUC: area under the ROC curve
CGC: Cancer Gene Census
CNV: copy number variation
DEGs: differentially expressed genes
DFS: disease‐free survival
ECOG: Eastern Cooperative Oncology Group
GEO: Gene Expression Omnibus
GWAS: genome‐wide association study
HMDD: Human microRNA Disease Database
HRD: homology recombination defect
NSCLC: nonsmall cell lung cancer
OS: overall survival
PFS: progression‐free survival
QQ: quantile‐quantile
ROC: receiver operating characteristic
SNP: single nucleotide polymorphisms
ssGSEA: single‐sample Gene Set Enrichment Analysis
TCGA: The Cancer Genome Atlas
TCR: T‐cell receptor
TF: transcription factor
TMB: tumor mutation burden
TMTI: T cell‐mediated tumor immune response

## Author Contributions

Hongsheng Guo conceived the study, curated the data, and drafted the original manuscript. Mengmeng Jiang performed the experiments, validated the methodology, and verified the results. Hailian Zhang provided resources, developed software, and created visualizations. Jian Zhao managed the project and supervised the work. Ying Luo conducted the data analysis, secured funding, revised the manuscript, and served as the corresponding author.

## Funding

This study was supported by the Tianjin Municipal Education Commission Research Program Project, 2024ZXZD019.

## Ethics Statement

Ethical approval was not required for this study because it is not involved any human or animal experiments.

## Consent

The authors have nothing to report.

## Conflicts of Interest

The authors have nothing to report.

## Supporting Information

Additional supporting information can be found online in the Supporting Information section.

## Supporting information


**Supporting Information 1** Figure S1: External evaluation of the eight‐gene score in pretreatment samples from the Durvalumab‐plus‐SBRT arm **of** GSE253564. (A) ROC curve for No_MPR versus MPR, with AUC and 95% confidence interval. (B) Score distributions by major pathologic response. No_MPR was treated as the positive class. The cohort‐derived Youden threshold is exploratory.

**Supporting Information 2** Figure S2: Abundance of immune cell infiltration in the resistant and sensitive groups defined by the PD‐1 treatment resistance score model in TCGA‐NSCLC. **(**A–G) Immune‐cell infiltration abundance was estimated using the ssGSEA algorithm. Panels illustrate distinct immune‐cell subtypes.

**Supporting Information 3** Figure S3: Immune microenvironment pattern mediated by anti‐PD‐1 therapy resistance score model. (A) The radar chart shows the proportion of CD8+ T cells identified by seven immune infiltration assessment algorithms in the PD‐1‐resistant and sensitive groups. (B) Heat map of differences in immune parameters between anti‐PD‐1 resistant and sensitive groups in TCGA‐NSCLC ( ^∗∗∗^: *p* < 0.001,  ^∗∗^: *p* < 0.01,  ^∗^: *p* < 0.05, Wilcoxon rank sum test). (C) Differences in TCR richness between anti‐PD‐1 resistance and sensitive groups in TCGA‐NSCLC. The middle line represents the median, and the upper and lower lines represent the upper and lower quartiles. (D) The heat map shows immune checkpoint gene expression between the anti‐PD‐1 treatment resistant and sensitive groups in TCGA‐NSCLC. (E) Correlation network of differentially expressed immune checkpoint genes and DEGs between the sensitive group and the resistant group.

**Supporting Information 4** Figure S4: Intratumor and intertumor heterogeneity of anti‐PD‐1 therapy resistance. **(**A) Identity of 33,694 cells annotated after clustering. (B) Bar graphs show the relative proportions of cell types in the five NSCLC samples. (C) Expression patterns of potential drivers of resistance to anti‐PD‐1 therapy across cell types. (D) Cell types distributed in sensitive patients and resistant patients. (E) The relative proportion of cells from the resistant and sensitive groups in each NSCLC patient.

**Supporting Information 5** Figure S5: Intercellular communication analysis reveals the resistance mechanism of anti‐PD‐1 treatment. **(**A) Comparison of ligand‐receptor interactions between PD1‐resistant and sensitive groups. Red represents the sensitive group having more communication than the resistant group, otherwise blue. B: Bars show the relative proportion of the interaction strength of each signaling pathway between the anti‐PD‐1 treatment resistant group and the sensitive group. The top red signal pathway is enriched in the resistant group, and the blue signal pathway is enriched in the sensitive group. (C,D) The ligand‐receptor pairs of cancer cells acting on immune cells and immune cells acting on cancer cells in the anti‐PD‐1 treatment resistant and sensitive groups. *p*‐values are expressed as dot size and communication probability as dot color.

**Supporting Information 6** Figure S6: Exploratory two‐sample MR reconstruction of *Bilophila wadsworthia* abundance and circulating ITGB6 protein levels. **(**A) SNP‐exposure versus SNP‐outcome effects and the two‐SNP IVW slope. (B) SNP‐specific Wald‐ratio estimates and 95% confidence intervals. (C) Manual leave‐one‐out estimates; each exclusion leaves one instrument and is descriptive. (D) Reverse Wald‐ratio estimate using the single independent ITGB6 trans‐pQTL. The forward and reverse *p* values are nominal and were not adjusted for the unavailable original 44 × 8 testing matrix.

**Supporting Information 7** Table S1: Overlapping genes between resistance associated DEGs and survival related genes at successive log₂ fold change cut offs.

**Supporting Information 8** Table S2: List of intestinal microbiota in the literature.

## Data Availability

The datasets generated and/or analyzed during the current study are available in the [GSE126045] repository, [https://www.ncbi.nlm.nih.gov/geo/query/acc.cgi?acc=GSE126045], [GSE41271] repository, [https://www.ncbi.nlm.nih.gov/geo/query/acc.cgi?acc=GSE41271] and [GSE68465] repository, [https://www.ncbi.nlm.nih.gov/geo/query/acc.cgi?acc=GSE68465].
