# Supplementary material for: Shining a Light on the Mechanisms of Drug Resistance to Anti‐PD‐1 Therapy in Non‐small Cell Lung Cancer
Source: Hum Mutat. 2026 Oct 28;2026:7216680. doi: 10.1155/humu/7216680 (PMC13717238; doi:10.1155/humu/7216680)
Supplement: Supplementary file 1 [file HUMU-2026-7216680-s001.pdf]

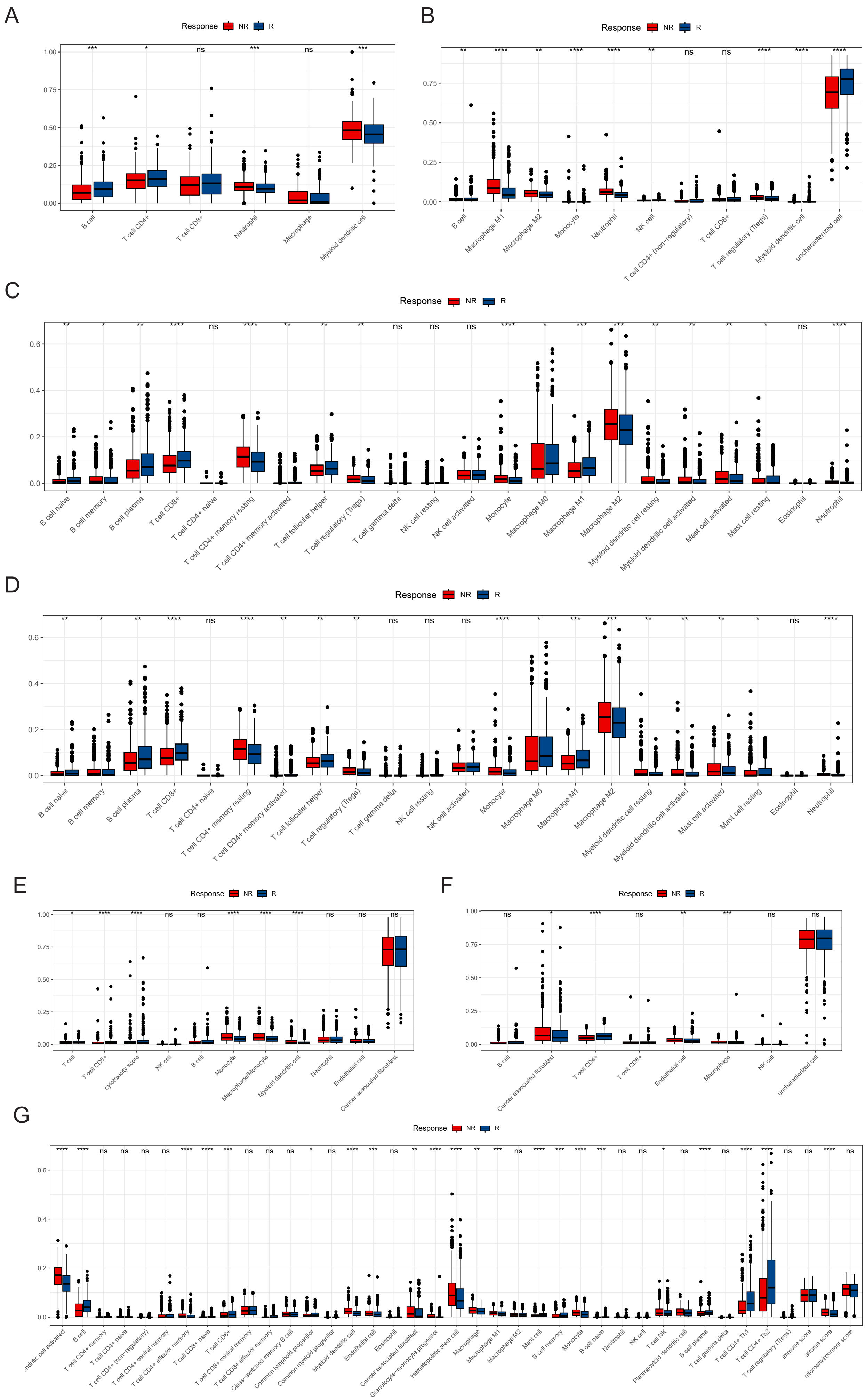

A
Response NR R
B cell
T cell CD4+
T cell CD8+
Neutrophil
Macrophage
Myeloid dendritic cell
B
Response NR R
B cell
Macrophage M1
Macrophage M2
Monocyte
Neutrophil
NK cell
T cell CD4+ (non−regulatory)
T cell CD8+
T cell regulatory (Tregs)
Myeloid dendritic cell
uncharacterized cell
C
Response NR R
B cell naive
B cell memory
B cell plasma
T cell CD8+
T cell CD4+ naive
T cell CD4+ memory resting
T cell CD4+ memory activated
T cell follicular helper
T cell regulatory (Tregs)
T cell gamma delta
NK cell resting
NK cell activated
Monocyte
Macrophage M0
Macrophage M1
Macrophage M2
Myeloid dendritic cell resting
Myeloid dendritic cell activated
Mast cell activated
Mast cell resting
Eosinophil
Neutrophil
D
Response NR R
E
Response NR R
T cell
T cell CD8+
cytotoxicity score
NK cell
B cell
Monocyte
Macrophage/Monocyte
Myeloid dendritic cell
Neutrophil
Endothelial cell
Cancer associated fibroblast
F
Response NR R
B cell
Cancer associated fibroblast
T cell CD4+
T cell CD8+
Endothelial cell
Macrophage
NK cell
uncharacterized cell
G
Response NR R
B cell
T cell CD4+ memory
T cell CD4+ naive
T cell CD4+ (non-regulatory)
T cell CD4+ central memory
T cell CD4+ effector memory
T cell CD8+ naive
T cell CD8+
T cell CD8+ central memory
T cell CD8+ effector memory
Class-switched memory B cell
Common lymphoid progenitor
Common myeloid progenitor
Myeloid dendritic cell
Endothelial cell
Eosinophil
Cancer associated fibroblast
Granulocyte-monocyte progenitor
Hematopoietic stem cell
Macrophage
Macrophage M1
Macrophage M2
Mast cell
B cell memory
Monocyte
B cell naive
Neutrophil
NK cell
T cell NK
Plasmacytoid dendritic cell
B cell plasma
T cell gamma delta
T cell CD4+ Th1
T cell CD4+ Th2
T cell regulatory (Tregs)
immune score
stroma score
microenvironment score
