# Supplementary material for: Shining a Light on the Mechanisms of Drug Resistance to Anti‐PD‐1 Therapy in Non‐small Cell Lung Cancer
Source: Hum Mutat. 2026 Oct 28;2026:7216680. doi: 10.1155/humu/7216680 (PMC13717238; doi:10.1155/humu/7216680)
Supplement: Supplementary file 2 [file HUMU-2026-7216680-s002.pdf]

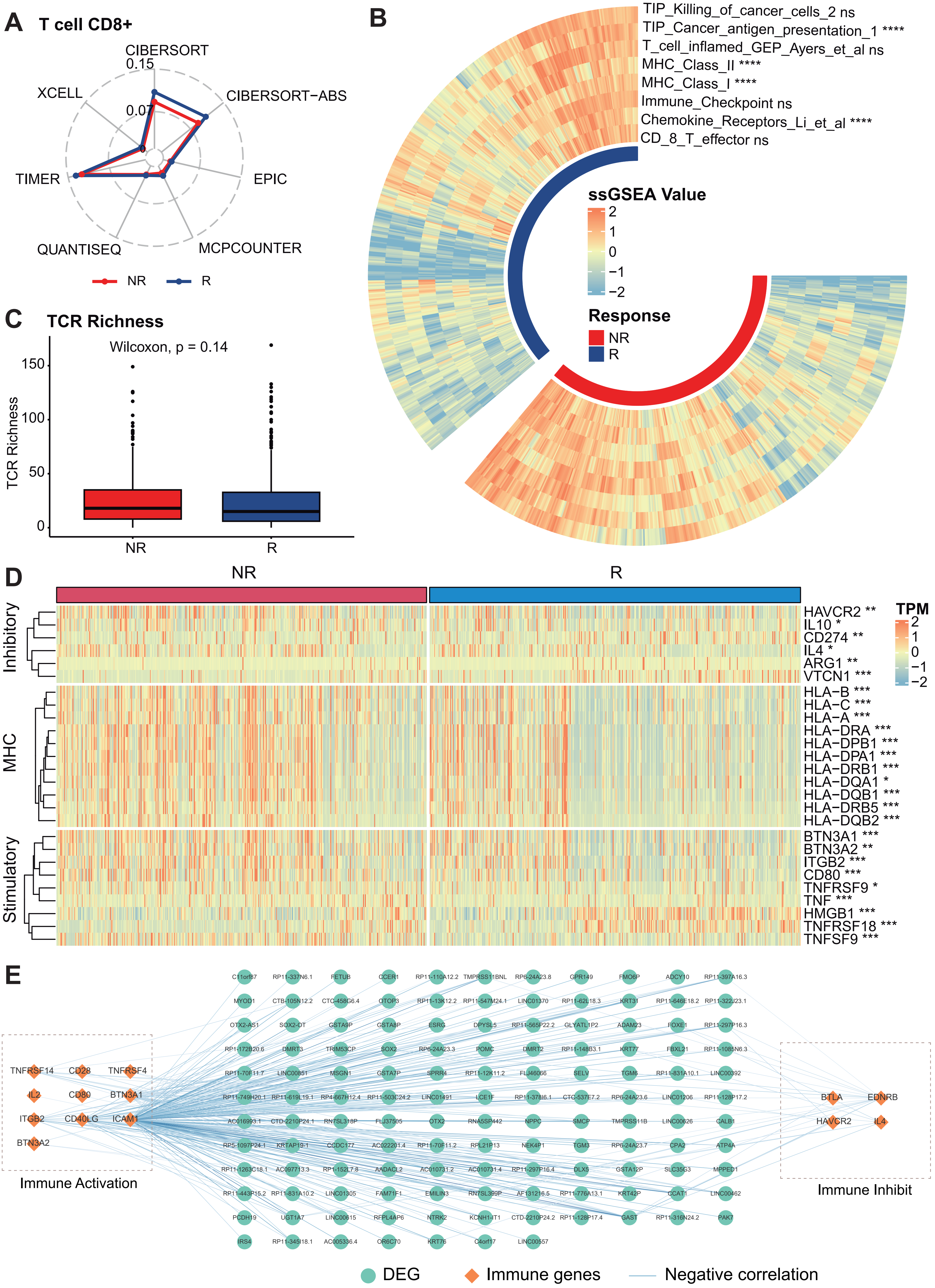
A
T cell CD8+
CIBERSORT
0.15
0.07
0
CIBERSORT−ABS
EPIC
MCPCOUNTER
QUANTISEQ
TIMER
XCELL
NR
R
B
TIP_Killing_of_cancer_cells_2 ns
TIP_Cancer_antigen_presentation_1 ****
T_cell_inflamed_GEP_Ayers_et_al ns
MHC_Class_II ****
MHC_Class_I ****
Immune_Checkpoint ns
Chemokine_Receptors_Li_et_al ****
CD_8_T_effector ns
ssGSEA Value
2
1
0
−1
−2
Response
NR
R
C
TCR Richness
Wilcoxon, p = 0.14
TCR Richness
150
100
50
0
NR
R
D
NR
R
Inhibitory
MHC
Stimulatory
HAVCR2 **
IL10 *
CD274 **
IL4 *
ARG1 **
VTCN1 ***
HLA−B ***
HLA−C ***
HLA−A ***
HLA−DRA ***
HLA−DPB1 ***
HLA−DPA1 ***
HLA−DRB1 ***
HLA−DQA1 *
HLA−DQB1 ***
HLA−DRB5 ***
HLA−DQB2 ***
BTN3A1 ***
BTN3A2 **
ITGB2 ***
CD80 ***
TNFRSF9 *
TNF ***
HMGB1 ***
TNFRSF18 ***
TNFSF9 ***
TPM
2
1
0
−1
−2
E
TNFRSF14
CD28
TNFRSF4
IL2
CD80
BTN3A1
ITGB2
CD40LG
ICAM1
BTN3A2
Immune Activation
BTLA
EDNRB
HAVCR2
IL4
Immune Inhibit
DEG
Immune genes
Negative correlation
