# Supplementary material for: Shining a Light on the Mechanisms of Drug Resistance to Anti‐PD‐1 Therapy in Non‐small Cell Lung Cancer
Source: Hum Mutat. 2026 Oct 28;2026:7216680. doi: 10.1155/humu/7216680 (PMC13717238; doi:10.1155/humu/7216680)
Supplement: Supplementary file 3 [file HUMU-2026-7216680-s004.pdf]

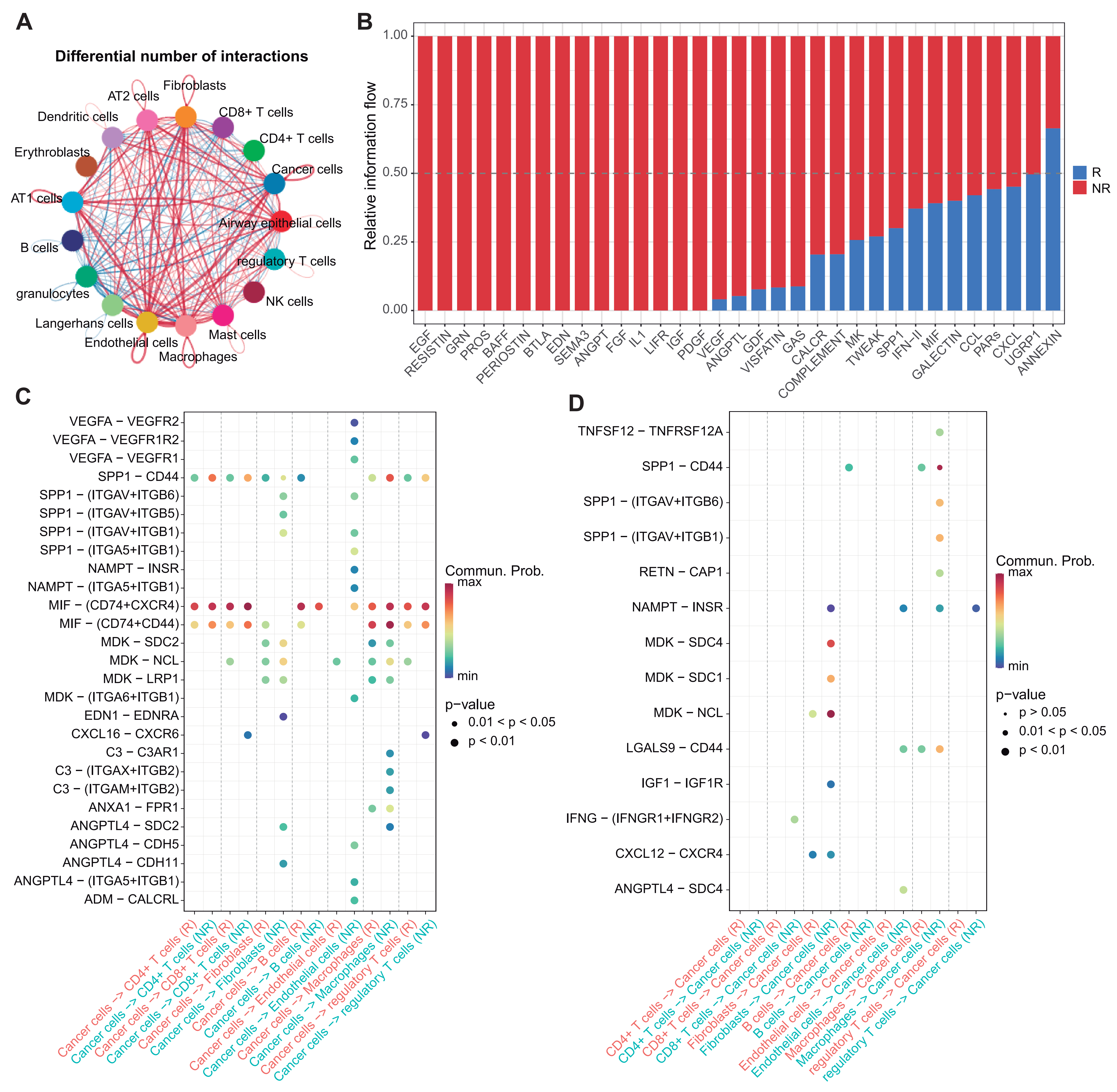
A
Differential number of interactions
Fibroblasts
AT2 cells
CD8+ T cells
Dendritic cells
CD4+ T cells
Erythroblasts
Cancer cells
AT1 cells
Airway epithelial cells
B cells
regulatory T cells
granulocytes
NK cells
Langerhans cells
Mast cells
Endothelial cells
Macrophages
B
Relative information flow
1.00
0.75
0.50
0.25
0.00
R
NR
EGF
RESISTIN
GRN
PROS
BAFF
PERIOSTIN
BTLA
EDN
SEMA3
ANGPT
FGF
IL1
LIFR
IGF
PDGF
VEGF
ANGPTL
GDF
VISFATIN
GAS
CALCR
COMPLEMENT
MK
TWEAK
SPP1
IFN−II
MIF
GALECTIN
CCL
PARs
CXCL
UGRP1
ANNEXIN
C
VEGFA − VEGFR2
VEGFA − VEGFR1R2
VEGFA − VEGFR1
SPP1 − CD44
SPP1 − (ITGAV+ITGB6)
SPP1 − (ITGAV+ITGB5)
SPP1 − (ITGAV+ITGB1)
SPP1 − (ITGA5+ITGB1)
NAMPT − INSR
NAMPT − (ITGA5+ITGB1)
MIF − (CD74+CXCR4)
MIF − (CD74+CD44)
MDK − SDC2
MDK − NCL
MDK − LRP1
MDK − (ITGA6+ITGB1)
EDN1 − EDNRA
CXCL16 − CXCR6
C3 − C3AR1
C3 − (ITGAX+ITGB2)
C3 − (ITGAM+ITGB2)
ANXA1 − FPR1
ANGPTL4 − SDC2
ANGPTL4 − CDH5
ANGPTL4 − CDH11
ANGPTL4 − (ITGA5+ITGB1)
ADM − CALCRL
Commun. Prob.
max
min
p−value
0.01 < p < 0.05
p < 0.01
Cancer cells −> CD4+ T cells (R)
Cancer cells −> CD4+ T cells (NR)
Cancer cells −> CD8+ T cells (R)
Cancer cells −> CD8+ T cells (NR)
Cancer cells −> Fibroblasts (R)
Cancer cells −> Fibroblasts (NR)
Cancer cells −> B cells (R)
Cancer cells −> B cells (NR)
Cancer cells −> Endothelial cells (R)
Cancer cells −> Endothelial cells (NR)
Cancer cells −> Macrophages (R)
Cancer cells −> Macrophages (NR)
Cancer cells −> regulatory T cells (R)
Cancer cells −> regulatory T cells (NR)
D
TNFSF12 − TNFRSF12A
SPP1 − CD44
SPP1 − (ITGAV+ITGB6)
SPP1 − (ITGAV+ITGB1)
RETN − CAP1
NAMPT − INSR
MDK − SDC4
MDK − SDC1
MDK − NCL
LGALS9 − CD44
IGF1 − IGF1R
IFNG − (IFNGR1+IFNGR2)
CXCL12 − CXCR4
ANGPTL4 − SDC4
Commun. Prob.
max
min
p−value
p > 0.05
0.01 < p < 0.05
p < 0.01
CD4+ T cells −> Cancer cells (R)
CD4+ T cells −> Cancer cells (NR)
CD8+ T cells −> Cancer cells (R)
CD8+ T cells −> Cancer cells (NR)
Fibroblasts −> Cancer cells (R)
Fibroblasts −> Cancer cells (NR)
B cells −> Cancer cells (R)
B cells −> Cancer cells (NR)
Endothelial cells −> Cancer cells (R)
Endothelial cells −> Cancer cells (NR)
Macrophages −> Cancer cells (R)
Macrophages −> Cancer cells (NR)
regulatory T cells −> Cancer cells (R)
regulatory T cells −> Cancer cells (NR)
