# Supplementary material for: Shining a Light on the Mechanisms of Drug Resistance to Anti‐PD‐1 Therapy in Non‐small Cell Lung Cancer
Source: Hum Mutat. 2026 Oct 28;2026:7216680. doi: 10.1155/humu/7216680 (PMC13717238; doi:10.1155/humu/7216680)
Supplement: Supplementary file 4 [file HUMU-2026-7216680-s005.pdf]

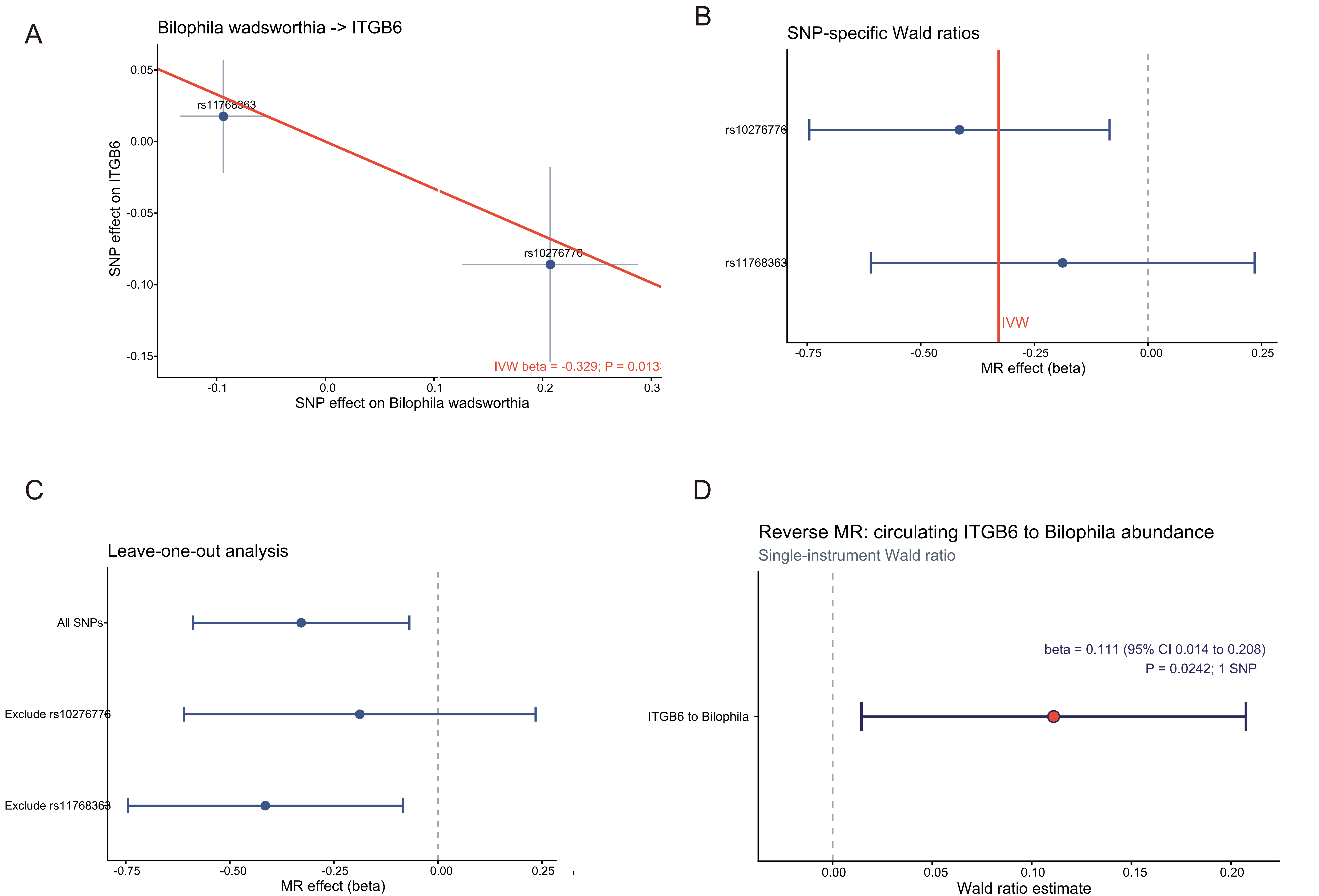

A
Bilophila wadsworthia -> ITGB6
SNP effect on ITGB6
SNP effect on Bilophila wadsworthia
rs11768363
rs10276776
IVW beta = -0.329; P = 0.013
0.05
0.00
-0.05
-0.10
-0.15
-0.1
0.0
0.1
0.2
0.3
B
SNP-specific Wald ratios
rs10276776
rs11768363
IVW
MR effect (beta)
-0.75
-0.50
-0.25
0.00
0.25
C
Leave-one-out analysis
All SNPs
Exclude rs10276776
Exclude rs11768363
MR effect (beta)
-0.75
-0.50
-0.25
0.00
0.25
D
Reverse MR: circulating ITGB6 to Bilophila abundance
Single-instrument Wald ratio
beta = 0.111 (95% CI 0.014 to 0.208)
P = 0.0242; 1 SNP
ITGB6 to Bilophila
Wald ratio estimate
0.00
0.05
0.10
0.15
0.20
