# Supplementary material for: Shining a Light on the Mechanisms of Drug Resistance to Anti‐PD‐1 Therapy in Non‐small Cell Lung Cancer
Source: Hum Mutat. 2026 Oct 28;2026:7216680. doi: 10.1155/humu/7216680 (PMC13717238; doi:10.1155/humu/7216680)
Supplement: Supplementary file 5 [file HUMU-2026-7216680-s007.pdf]

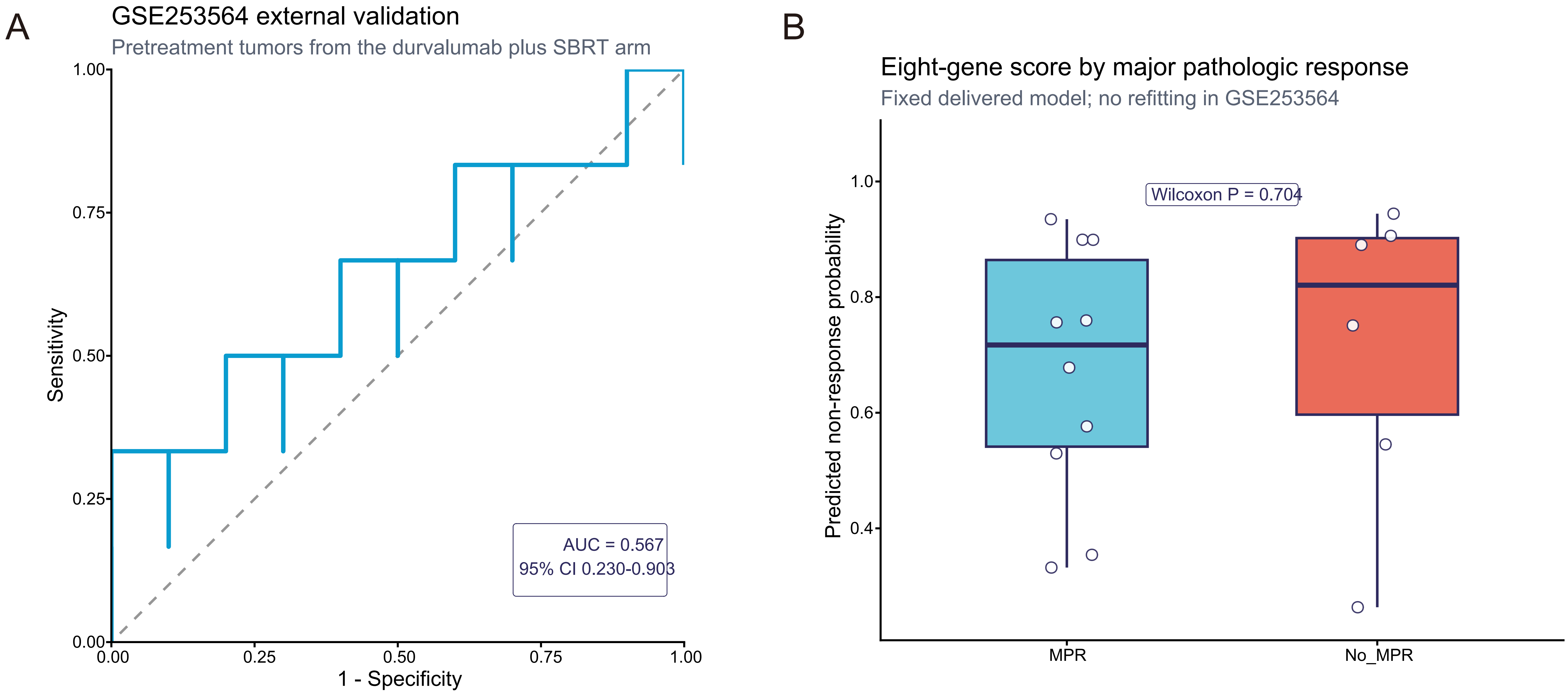
A
GSE253564 external validation
Pretreatment tumors from the durvalumab plus SBRT arm
Sensitivity
1 - Specificity
0.00
0.25
0.50
0.75
1.00
AUC = 0.567
95% CI 0.230-0.903
B
Eight-gene score by major pathologic response
Fixed delivered model; no refitting in GSE253564
Predicted non-response probability
0.4
0.6
0.8
1.0
Wilcoxon P = 0.704
MPR
No_MPR
