# Supplementary material for: Shining a Light on the Mechanisms of Drug Resistance to Anti‐PD‐1 Therapy in Non‐small Cell Lung Cancer
Source: Hum Mutat. 2026 Oct 28;2026:7216680. doi: 10.1155/humu/7216680 (PMC13717238; doi:10.1155/humu/7216680)
Supplement: Supplementary file 6 [file HUMU-2026-7216680-s008.pdf]

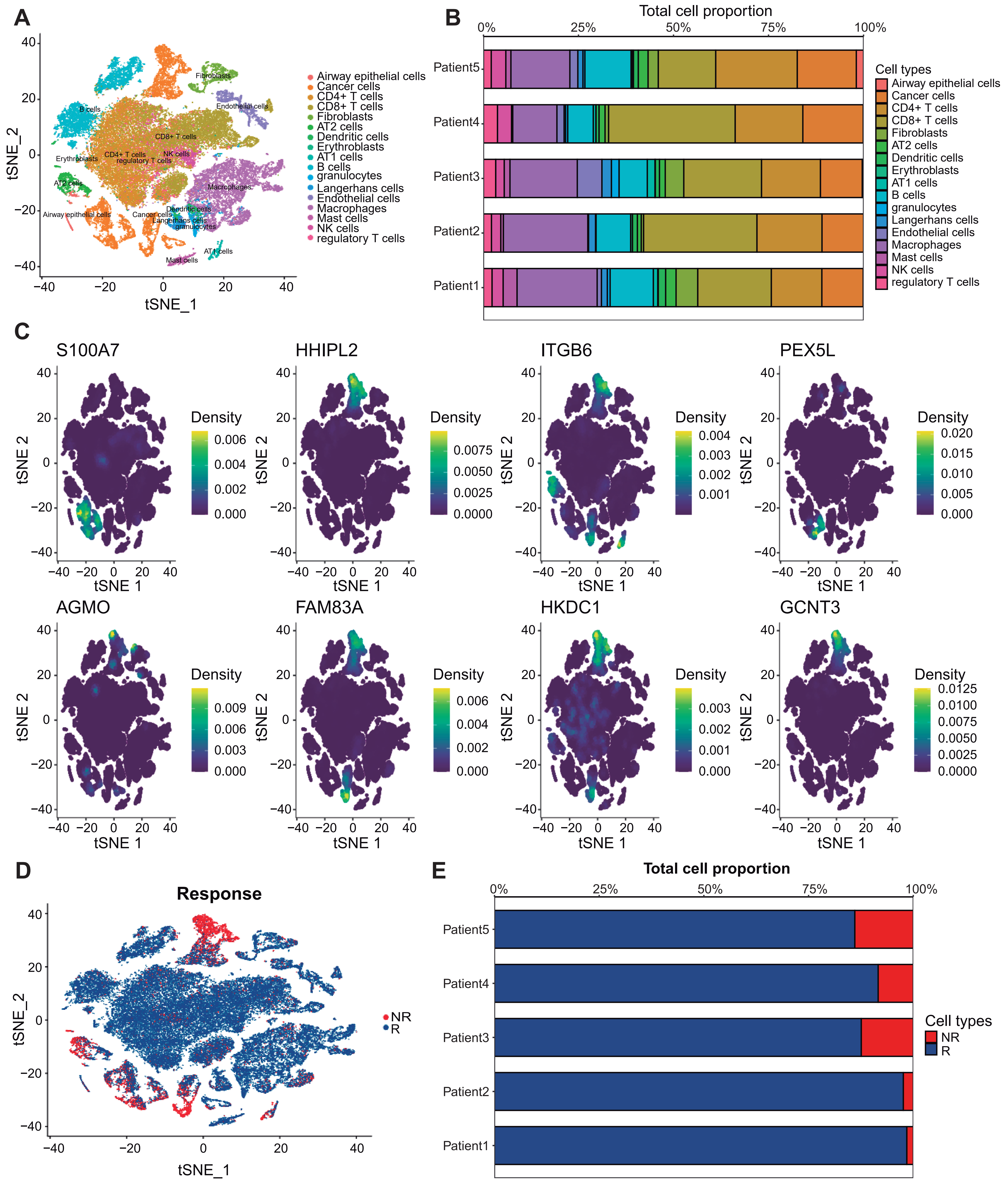

A
B
Total cell proportion
0%
25%
50%
75%
100%
Patient5
Patient4
Patient3
Patient2
Patient1
Cell types
Airway epithelial cells
Cancer cells
CD4+ T cells
CD8+ T cells
Fibroblasts
AT2 cells
Dendritic cells
Erythroblasts
AT1 cells
B cells
granulocytes
Langerhans cells
Endothelial cells
Macrophages
Mast cells
NK cells
regulatory T cells
tSNE_1
tSNE_2
C
S100A7
HHIPL2
ITGB6
PEX5L
AGMO
FAM83A
HKDC1
GCNT3
Density
tSNE 1
tSNE 2
D
Response
NR
R
E
Total cell proportion
Cell types
NR
R
