# Supplementary material for: Shining a Light on the Mechanisms of Drug Resistance to Anti‐PD‐1 Therapy in Non‐small Cell Lung Cancer
Source: Hum Mutat. 2026 Oct 28;2026:7216680. doi: 10.1155/humu/7216680 (PMC13717238; doi:10.1155/humu/7216680)
Supplement: Supplementary file 7 [file HUMU-2026-7216680-s006.docx]

| Log2FC threshold | DEG | DEG∩ Survival gene | 8-gene | New candidate |
| --- | --- | --- | --- | --- |
| 1 | 101 | 17 | 8/8 | COL4A3, SLC26A5, FAM111B, KRT80, PAWR, FRMD5, EMP2, TMC7, ZNF544 |
| 1.5 | 81 | 15 | 8/8 | COL4A3, SLC26A5, FAM111B, KRT80, PAWR, FRMD5, TMC7 |
| 2 | 62 | 13 | 8/8 | COL4A3, SLC26A5, FAM111B, KRT80, FRMD5 |
| 2.5 | 56 | 10 | 8/8 | FAM111B, KRT80 |
| 3 | 42 | 8 | 8/8 | / |

**Table S1. Overlapping genes between resistance‑associated DEGs and survival‑related genes at successive log₂ fold‑change cut‑offs.**
